# Effect of rosuvastatin and ezetimibe therapy on LDL-cholesterol reduction and non-invasive arterial injury markers in children with heterozygous familial hypercholesterolemia: a prospective, open-label, randomized study

**DOI:** 10.3389/fcvm.2026.1933757

**Published:** 2026-09-29

**Authors:** Ajda Skarlovnik, Tevz Gorjanc, Jaka Sikonja, Jan Kafol, Matej Mlinaric, Barbara Cugalj Kern, Ana Drole Torkar, Jernej Kovac, Chiara Rodaro, Tadej Battelino, Urh Groselj

**Affiliations:** 1 Department of Vascular Diseases, Division of Internal Medicine, University Medical Centre Ljubljana, Ljubljana, Slovenia; 2 Faculty of Medicine, University of Ljubljana, Ljubljana, Slovenia; 3 Department of Endocrinology, Diabetes, and Metabolic Diseases, University Children's Hospital, University Medical Centre Ljubljana, Ljubljana, Slovenia; 4 Department of Endocrinology, Diabetes and Metabolic Diseases, Division of Internal Medicine, University Medical Centre Ljubljana, Ljubljana, Slovenia; 5 Clinical Institute of Special Laboratory Diagnostics, University Children's Hospital, University Medical Centre Ljubljana, Ljubljana, Slovenia; 6 Center for Rare Diseases, University Children's Hospital, University Medical Centre Ljubljana, Ljubljana, Slovenia; 7 Clinical Department of Medical, Surgical and Health Science, University of Trieste, Trieste, Italy

**Keywords:** arterial injury, familial hypercholesterolemia, lipid lower medications, pediatric, preventive medicine, statins

## Abstract

**Background and aims:**

Individuals with familial hypercholesterolemia (FH) require early lipid-lowering therapy (LLT). We compared rosuvastatin versus ezetimibe treatment and the role of major non-invasive arterial injury markers in children with heterozygous FH.

**Methods:**

In this prospective, open-label, randomized study, 45 children with FH [median age 10.5 years (IQR 8.7–14.5)] received rosuvastatin 5 mg OD or ezetimibe 10 mg OD for 6.2 months. Carotid intima-media thickness (cIMT), reactive hyperemia index (RHI), pulse wave velocity (PWV), and beta stiffness were evaluated in relation to anthropometric and lipid-related variables.

**Results:**

Rosuvastatin produced a greater LDL-C reduction than ezetimibe (36.7% vs. 18.3%; *p* < 0.001) with 9/20 (45%) children achieving LDL-C <3.5 mmol/L compared to 2/15 (13%) in ezetimibe group. No reports of myalgias, elevations in transaminases, or creatine kinase (CK) were observed in any of the groups. Markers at baseline correlated mainly with age and anthropometry. Beta stiffness decreased during LLT in the overall cohort (−0.24; 95% CI −0.51 to −0.03, *p* = 0.002) and in the rosuvastatin group (−0.4; 95% CI −0.7 to −0.1, *p* = 0.028), with no between-group difference (*p* = 0.266). This likely reflects regression to the mean, as LDL-C reduction and treatment allocation had only small effects across all markers.

**Conclusions:**

Rosuvastatin achieved greater LDL-C reduction than ezetimibe in children with FH, without difference in safety and tolerability profiles. Arterial injury markers correlated mainly with age and anthropometry, and their short-term changes were not treatment-related, likely due to limited power and the short follow-up.

## 1 Introduction

Heterozygous familial hypercholesterolaemia (HeFH) is a common monogenic disorder characterised by markedly elevated low-density lipoprotein cholesterol (LDL-C) since birth, leading to accelerated atherosclerotic process and an increased lifetime risk of premature cardiovascular disease (CVD) in untreated individuals (1–3). Early recognition is crucial, as effective treatment can reduce the lifelong disease burden; however, since FH in childhood is often subclinical, relying solely on lipid parameters may be insufficient, thus non-invasive and cost-effective measures of arterial injury can be used in this period to monitor CVD burden (4–6). Currently, well-established methods for assessing early arterial injury include the evaluation of vessel wall thickness using carotid intima–media thickness (cIMT), endothelial function using the reactive hyperaemia index (RHI), and arterial stiffness using pulse wave velocity (PWV) and beta stiffness (7–9). Although these methods have been extensively studied, the findings remain inconsistent, particularly in paediatric populations, where normal growth and development exert a strong influence (8, 10).

The study aimed to (i) compare the LDL-C–lowering effects and safety profiles of rosuvastatin and ezetimibe in children with FH, (ii) to evaluate the influence of lipid- and growth-related factors on arterial injury markers, iii) and to determine whether non-invasive arterial injury markers reflect short-term vascular changes.

## 2 Methods

### 2.1 Study design and cohort selection

We assessed the eligibility of 61 white Caucasian children and adolescents with clinical FH, identified through the national FH screening programme and managed between June 2016 and September 2020 at the Department of Endocrinology, Diabetes, and Metabolism, University Children's Hospital, University Medical Center Ljubljana (UCHL-UMC) (11). Informed consent was obtained from the study participants or their parents/caregivers to conduct this study and to publish the results from clinical and genetic analyses. The primary inclusion criteria were: (i) presence of a likely pathogenic or pathogenic variant in one of the three main genes associated with FH (*LDLR*, *APOB*, or *PCSK9*); (ii) at least one measurement of non-invasive markers of arterial injury; (iii) age under 18 years. The main exclusion criteria were: (i) prior use of lipid-lowering therapy; (ii) inadequate quality of arterial marker measurements; (iii) diagnosis of compound heterozygous or homozygous FH; (iv) identification of a variant of uncertain significance (VUS) in FH-related or other genes; (v) presence of other rare dyslipidaemias, such as lysosomal acid lipase deficiency or lipoprotein lipase deficiency; (vi) presence of other major chronic illnesses that could cause arterial injury; (vii) refusal of treatment initiation by patients or their parents/caregivers.

This was a prospective, open-label, randomised interventional study comprising three study visits (V0, V1, and V2), as shown in Figure 1. As the aim of the study was not to demonstrate efficacy against no treatment but to compare the magnitude of LDL-C reduction and short-term safety and tolerability between two standard-of-care therapies, an untreated control group was unnecessary. The trial was conducted as an open-label study because the two medications differ in appearance and dosing, making blinding impractical, and because the primary outcomes—lipid measurements and vascular parameters—are objective and unlikely to be influenced by awareness of treatment allocation. At baseline (V0), participants underwent a medical history review, physical examination, blood pressure and anthropometric measurements, venous blood sampling, and non-invasive assessment of arterial injury markers (cIMT, RHI, PWV, and beta stiffness). Randomisation was performed at V0 using simple randomisation to assign participants to either the rosuvastatin (5 mg once daily) or ezetimibe (10 mg once daily) group. All included children were advised to follow a healthy, balanced diet with limited intake of animal-derived fats and adequate consumption of vegetables and polyunsaturated fats. Dietary intake and adherence to these recommendations were not systematically assessed. Participants and investigators were not masked to treatment allocation, except for the investigator performing arterial injury marker measurements. The baseline visit (V0) was followed by follow-up visits at 3 months (V1) and 6 months (V2). V1 included only venous blood sampling, while at V2 all baseline assessments were repeated. Changes across all three visits were then analysed. A Central Illustration was created to summarize the study design and main findings.

**Figure 1:**
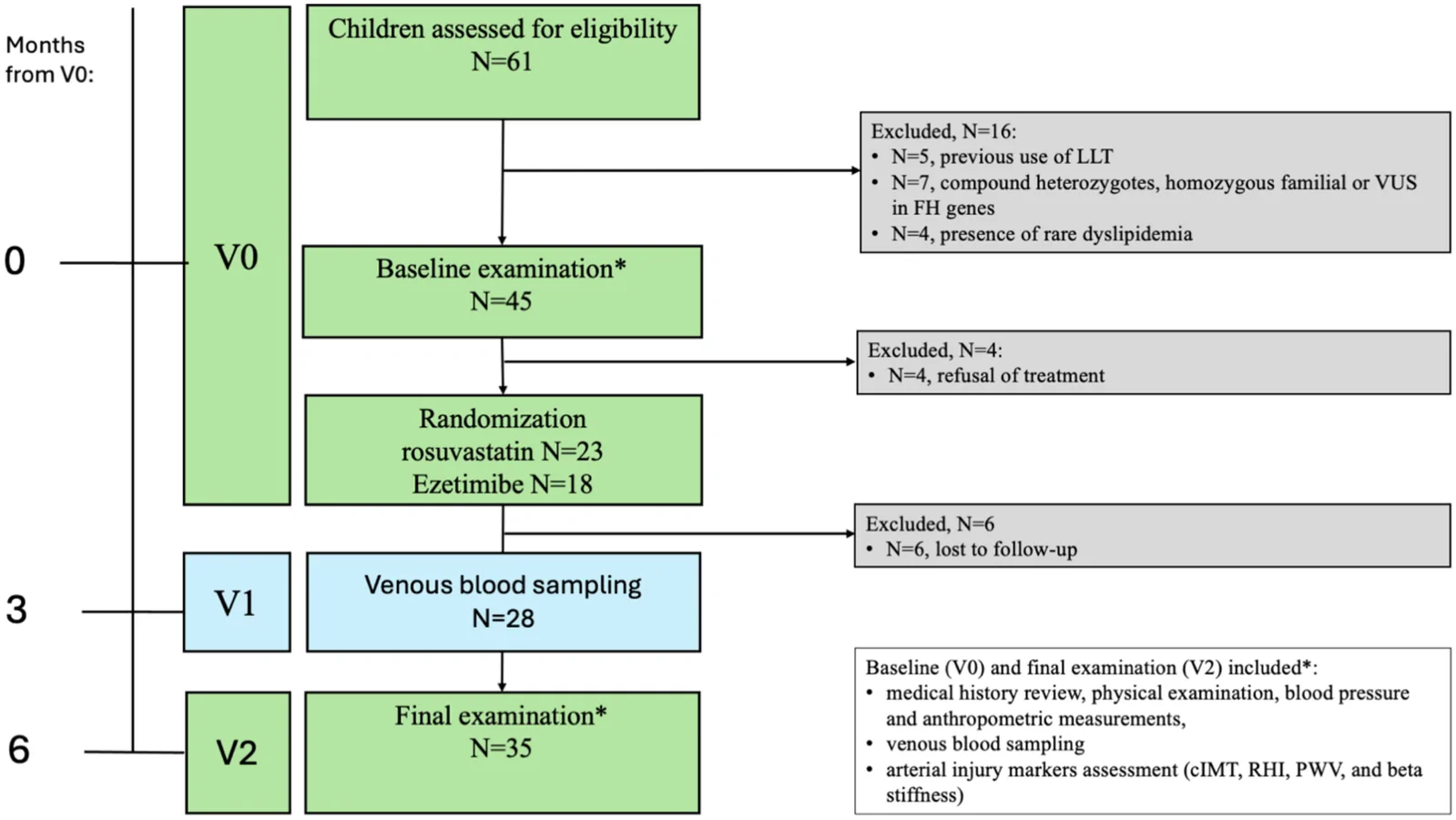
Study flowchart. N, number of patients; V, visit; LLT, lipid-lowering therapy; VUS, variant of uncertain significance.

### 2.2 Measurements

All measurements were performed in a standardised, comfortable environment and under the same conditions at each visit. Clinical history and standard clinical examination were conducted by a paediatric lipidologist. Anthropometric parameters were measured using standard methods. As body weight and height increase with age, we used *z*-scores to account for growth-related differences. Anthropometric *z*-scores were calculated from UK-WHO Growth Charts using the Microsoft Excel extension LMSGrowth (available at: https://www.healthforallchildren.com/shop-base/shop/software/lmsgrowth/) (12). Blood pressure was measured using a validated oscillometric device. Blood samples were collected in the early morning after an overnight fast. Cumulative TC (TC Exp), ApoB (ApoB Exp), and LDL-C (LDL-C Exp) exposures were calculated by multiplying serum TC, ApoB, or LDL-C (in mmol/L) at V1 by the patient's age (in years) at V1. Elevated Lp(a) levels above 50 mg/dL were considered a risk modifier for atherosclerotic cardiovascular disease (ASCVD) (13). Treatment response was classified as sufficient if LDL-C at V2 was <3.5 mmol/L (14). Safety assessments included elevations in transaminases (ALT and AST) and creatine kinase (CK), defined as ≥2× the upper limit of normal for transaminases and ≥3.5× for CK.

### 2.3 Ultrasound measurements of the carotid intima media thickness and arterial stiffness

Ultrasound measurements were performed by a single investigator who was blinded to the patients’ treatment status and did not have access to previous scans during follow-up assessments. The examinations were conducted using the Aloka 5500 SSD Pro-sound ultrasound system (Aloka Co., Ltd., Tokyo, Japan) with a UST-5539 (7.5 MHz) linear probe, in accordance with the manufacturer's guidelines. The Auto-cIMT modality was used, and the cIMT value, reported in millimetres, was calculated as the average of six bilateral measurements. In addition, a high-definition echo-tracking system (Aloka E-tracking software) was used to generate the artery's pressure–diameter curve. Beta stiffness was calculated using the following equation: beta stiffness = ln (Ps/Pd)/[(Ds−Dd)/Dd], where Ps = systolic BP, Pd = diastolic BP, Ds = arterial systolic diameter, and Dd = arterial diastolic diameter. Pulse wave velocity (PWV) was calculated from the time delay between the two adjacent distension waveforms of the water hammer equation for forward travelling waves using the beta stiffness.

### 2.4 Measurement of endothelial function

Endothelial function was determined from RHI and measured using the EndoPAT 2000 device (Itamar Medical Ltd., Caesarea, Israel). RHI was calculated according to the manufacturer's algorithm, based on ratios between pulse wave amplitudes during the reactive hyperaemia and baseline phases. RHI values ≤1.67 indicate endothelial dysfunction, values between 1.67 and 2.00 represent a borderline (“grey zone”), and values ≥2.00 are considered normal. In the study, the RHI cut-off for endothelial dysfunction was set at 1.67.

### 2.5 Laboratory measurements

Plasma lipid levels were measured using standardised assays. All genetic analyses were performed at UCHL-UMC and are described in our previous publications (5).

### 2.6 Sample size and statistical power

This study was not based on a formal *a priori* sample-size calculation and finally 45 patients were included. *Post hoc* power for the correlation analyses showed the study had adequate statistical power (≥0.80) to detect only moderate to large correlations (approximately |*ρ*| ≥ 0.40). Smaller associations fell below the detectable threshold and could not be reliably identified. Consequently, non-significant or weak correlations should be interpreted with caution, as the absence of statistical significance may reflect limited power rather than a true lack of association.

*Post hoc* power calculations based on the model's residual variance, standard errors, and effect sizes indicated that the available sample (V0: *n* = 41; V1: *n* = 33; V2: *n* = 35) provided >0.99 power to detect the observed overall percentage reduction in LDL-C over time (partial *η*^2^ ≈ 0.65). Using these estimates, a sample size of ∼15 patients per treatment group would have been sufficient to achieve >90% power (*α* = 0.05) to detect a between-group LDL-C difference of ∼0.8–1.0 mmol/L. While difference between treatment groups in likelihood of target attainment had only ∼61%, and approximately 31 participants per group would be needed to achieve 80% power.

Due to the limited number of children with paired arterial injury measurements (cIMT, PWV, and beta stiffness: *n* = 34; RHI: *n* = 31) the models had sufficient power to detect only moderate intra-individual changes. Sample-size estimates were derived from the observed within-subject effect sizes and model residual variances, assuming *α* = 0.05 (two-tailed). Under these parameters, approximately 50–70 participants would be required to reliably assess short-term changes across all four arterial markers. In contrast, LDL-C reduction and treatment group effects on marker changes were very small; using partial *η*^2^ values and corresponding variance estimates, detecting effects of this magnitude would require substantially larger samples, generally in the range of ∼100–500 participants depending on the vascular marker.

### 2.7 Statistical analysis

All statistical analyses were conducted using R software version 3.6.0 (R Foundation for Statistical Computing, Vienna, Austria). Continuous variables were assessed for normality using the Shapiro–Wilk test and normal probability plots. As most arterial injury markers and the majority of lipid, demographic, and anthropometric parameters were not normally distributed, all variables are reported as median [interquartile range (IQR): first quartile–third quartile], with minimum and maximum values provided for selected markers.

Correlations between individual arterial injury markers and their associations with demographic variables (age, sex), anthropometric variables (body height, body weight, and their corresponding *z*-scores), and lipid variables (LDL-C, high-density lipoprotein cholesterol [HDL-C], apolipoprotein B [ApoB], total cholesterol [TC], triglycerides [TG], lipoprotein(a) [Lp(a)], and TC Exp, ApoB Exp and LDL-C Exp) at baseline (V0) were assessed using Spearman's rank correlation coefficient (*ρ*). All lipid variables, excluding exposure variables, were additionally adjusted for age and sex using partial Spearman rank correlation to obtain standardized (adjusted) association estimates. Corresponding two-sided *p*-values are reported. Statistical power for correlation analyses was estimated using Fisher's *z*-transformation (two-tailed *α* = 0.05) based on the actual sample size for each vascular parameter; only correlations with |*ρ*| ≥ 0.40 achieved adequate power (≥0.8), whereas smaller effects were considered underpowered.

Serum LDL-C was analyzed using a linear mixed-effects model for repeated measures. The dependent variable was LDL-C (mmol/L). Fixed effects included time (V0, V1, V2), treatment group (rosuvastatin or ezetimibe), baseline LDL-C, and the time-by-treatment interaction. A random intercept for participant accounted for within-subject correlation. The model was estimated by restricted maximum likelihood (REML) using a compound symmetry covariance structure, selected based on model fit criteria. Missing data were assumed to be missing at random, and all available observations were used without imputation, all subjects had baseline LDL-C measurements, participants with partial follow-up data were retained in the analysis, avoiding loss of power and reducing bias. Adjusted least-squares means and 95% confidence intervals (CIs) were estimated from the model. Absolute and percent reductions from baseline were obtained from model contrasts (V1–V0 and V2–V0). Between-group differences were tested with Tukey-adjusted pairwise comparisons, and the proportion of participants achieving LDL-C <3.5 mmol/L was compared with Fisher's exact test. A *post hoc* power analysis based on observed proportions (*α* = 0.05, two-sided) indicated that the study was adequately powered for continuous LDL-C outcomes but underpowered for the binary treatment target comparison. Model assumptions were verified by inspection of residual–fitted and Q–Q plots. Although the Shapiro–Wilk test indicated mild deviation from normality, residuals were symmetric and homoscedastic, supporting model validity.

Changes in vascular parameters (cIMT, PWV, RHI, and beta stiffness) were calculated as follow-up minus baseline values. For each marker, two prespecified ANCOVA models were constructed. The overall model evaluated the biological effect of LDL-C lowering and included LDL-C reduction together with the baseline value of the same vascular marker, which was entered as a covariate to adjust for pre-treatment differences and minimize regression-to-the-mean bias. To determine whether drug class contributed effects beyond LDL-C reduction, a second treatment model was fitted that included the same covariates with the addition of treatment group (rosuvastatin vs. ezetimibe) as a fixed factor. All analyses used complete cases, and no imputation was required. Model outputs included unstandardized coefficients (B), partial eta squared (*η*^2^), *p*-values, and observed power. Adjusted mean changes and 95% confidence intervals were obtained using the estimated marginal means (EMM) procedure. For pairwise comparisons between treatment groups, for within-group adjusted mean changes, statistical significance was inferred based on whether the 95% CI excluded zero; *p*-values were reported only for significant estimates.

## 3 Results

### 3.1 Baseline clinical characteristics

A total of 45 adolescents [25 (55.6%) females] with a median age of 10.5 years (IQR 8.7–14.5) met the inclusion criteria, and their baseline characteristics are presented in Table 1. Nineteen (42.2%) children were younger than 10 years of age and all included children had LDL-C greater than 3.5 mmol/L at V0. LDL-C was negatively correlated with age (*ρ* = 0.670, *p* < 0.001) and no correlation between LDL-C and BMI *z*-score (*p* = 0.862) was observed. The median cumulative LDL-C exposure was 57.9 mmol/L × years (IQR 51.3–65.7), with a maximum of 117.7 mmol/L × years and a minimum of 41.7 mmol/L × years. Lp(a) greater than 50 mg/dL was measured in 6 (16.7%) children. At baseline, one child had elevated transaminases (2%), and CK was normal in all participants.

**Table 1:** Baseline characteristics of the whole study population.

Variable	Rosuvastatin, *N* = 23	Ezetimibe, *N* = 18	Total, *N* = 45
Females, *n* (%)	14 (60.9%)	8 (44.4%)	25 (55.6%)
Age, years	9.7 (8.4–14.2)	11.0 (9.1–15.1)	10.5 (8.7–14.5)
Body mass, kg	39.1 (34.0–48.6)	36.3 (32.3–66.2)	36.8 (32.7–57.2)
Body mass *z*-score	0.56 (0.09–1.47)	0.41 (−0.63 to 2.16)	0.51 (−0.15 to 1.11)
Body height, cm	143.4 (133.1–161.3)	147.7 (139.2–168.5)	146 (134–166)
Body height *z*-score	0.48 (–0.20 to 1.16)	0.53 (−0.09 to 1.18)	0.45 (−0.39 to 1.52)
BMI	19.7 (16.7–21.6)	18.7 (16.0–23.4)	19.2 (16.1–21.7)
BMI *z*-score	0.54 (–0.16 to 1.60)	0.36 (−0.59 to 1.65)	0.39 (−0.39 to 1.58)
Systolic blood pressure, mmHg	112 (107–117)	113 (101–128)	114 (104–122)
Diastolic blood pressure, mmHg	59 (57–64)	62 (54–72)	61 (56–67)
TC, mmol/L	7.1 (6.6–8.1)	6.8 (6.5–8.0)	6.8 (6.5–8.2)
LDL-C, mmol/L	5.2 (4.5–6.2)	4.9 (4.5–5.7)	5.1 (4.5–6.2)
HDL-C, mmol/L	1.4 (1.4–1.6)	1.4 (1.3–1.6)	1.4 (1.2–1.6)
ApoA1, g/L	1.3 (1.2–1.5)	1.3 (1.2–1.5)	1.4 (1.3–1.5)
ApoB, g/L	0.8 (0.7–1.1)	0.9 (0.6–1.2)	1.3 (1.2–1.5)
TG, mmol/L	0.8 (0.7–1.0)	0.9 (0.8–1.2)	0.8 (0.7–1.1)
Lp(a), mg/L	224.0 (101.0–390.5)	126.0 (89.4–260.5)	150.5 (100.0–372.0)

Data are *N* (%) or median (first quartile–third quartile). BMI, body mass index; TC, total cholesterol; LDL-C, low-density lipoprotein cholesterol; HDL, high-density lipoprotein cholesterol; ApoA1, apolipoprotein A1; ApoB, apolipoprotein B; Lp(a), lipoprotein(a); TG, triglycerides.

Baseline characteristics of the rosuvastatin and ezetimibe groups were well balanced (Table 1), with no differences in age (*p* = 0.358), ApoB (*p* = 0.706), TC (*p* = 0.563), LDL-C (*p* = 0.519), or Lp(a) (*p* = 0.471).

At baseline (V0), several arterial injury measurements were of insufficient quality and were therefore excluded. At visit 1, we successfully obtained cIMT in 41 patients, PWV and beta stiffness in 40 patients, and RHI in 37 patients. Figure 2 presents the age and sex adjusted Spearman partial correlation coefficients between the arterial injury markers and standardized lipid variables at baseline. Unadjusted heatmap showing correlations between arterial injury markers and age, anthropometric and lipid variables at baseline is in the Supplementary Figure S1.

**Figure 2:**
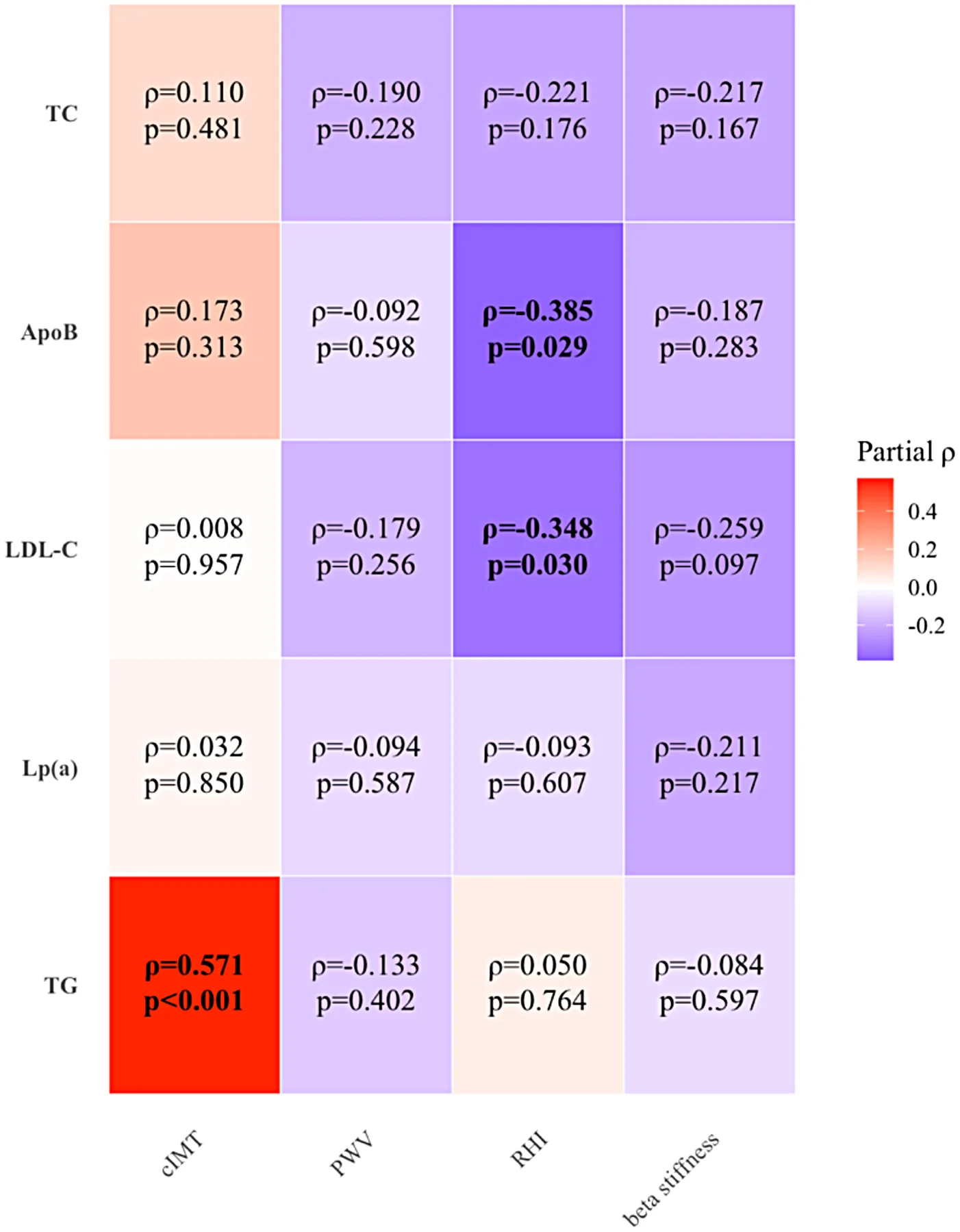
Correlation heatmap showing Spearman partial correlation coefficients between arterial injury markers and age and sex adjusted lipid characteristics at baseline (V0). Red colour indicates positive, while blue indicates negative correlation coefficient between listed variables; significant correlations are bolded (*p* < 0.05). cIMT, carotid intima-media thickness; PWV, pulse wave velocity; RHI, reactive hyperemia index; TC, total cholesterol; ApoB, apolipoprotein B; LDL-C, low-density lipoprotein cholesterol; Lp(a), lipoprotein(a); TG, triglycerides.

#### 3.1.1 Changes in LDL-C

Following randomisation at V0, a subset of participants attended an intermediate examination (V1) at a median of 2.3 months (IQR 1.8–2.9) and a final visit (V2) at a median of 6.2 months (IQR 6.0–6.7) after V0. Median body height increased by 4.03 cm (IQR 2.50–3.40; *p* < 0.001), median body weight by 3.70 kg (IQR 2.50–4.22; *p* < 0.001), and median BMI by 0.47 kg/m^2^ (IQR 0.00–0.98; *p* = 0.002). No significant changes were observed in height, weight, or BMI *z*-scores (*p* = 0.874, 0.274, and 0.094, respectively).

Although dropout among children included in the LDL-C reduction analysis was substantial (V1: *n* = 45; V2: *n* = 35; 22%), the pattern did not appear differential or likely to introduce bias. Boys had a numerically higher dropout rate than girls [5/19 (26.3%) vs. 2/22 (9.1%); *p* = 0.219], and dropout rates were comparable between the rosuvastatin and ezetimibe groups [3 (13%) vs. 4 (22%); *p* = 0.679]. Children lost to follow-up did not differ from those who completed V2 in baseline LDL-C (20.3 vs. 21.1 mmol/L; *p* = 0.900) or height *z*-score (*p* = 0.212), although they had slightly higher baseline weight and BMI *z*-scores (*p* = 0.044 and *p* = 0.032, respectively).

For the entire cohort, the median LDL-C level at visit V2 was 4.0 mmol/L (IQR 3.2–4.4). The mixed-effects model showed good fit (REML = 209.7), with between-subject variance of 0.010 and residual variance of 0.397 mmol^2^/L. Both the duration of short-term LLT (*p* < 0.001; *η*^2^ = 0.69) and the treatment-by-time interaction between study visits (*p* = 0.001; *η*^2^ = 0.21) were independent predictors of LDL-C change, with rosuvastatin showing faster and substantially greater LDL-C reductions at both follow-up visits compared with ezetimibe.

Model-adjusted mean LDL-C levels (mmol/L, 95 CI) were 5.4 mmol/L (95 CI 5.1–5.7) at V0, 3.2 (95 CI 2.9–3.5) at V1, and 3.4 mmol/L (3.1–3.7) at V2 for rosuvastatin, compared with 5.3 mmol/L (95 CI 5.0–5.6), 4.3 mmol/L (95 CI 3.9–4.6), and 4.4 mmol/L (95 CI 4.0–4.7) for ezetimibe. From baseline, rosuvastatin reduced LDL-C by −2.2 mmol/L [95 CI −2.7 to −1.7; −41.1% (95 CI −49.9 to −32.2)] at V1 and −2.0 mmol/L [95 CI −2.4 to −1.5; −36.7% (95 CI −44.9 to −28.6)] at V2. Reductions in LDL-C in each treatment group are presented in Figure 3. In comparison, ezetimibe achieved smaller reductions of −1.1 mmol/L [95 CI −1.6 to −0.6; −20.2% (95 CI −30.0 to −10.4)] at V1 and −1.0 mmol/L [95 CI −1.5 to −0.5; −18.3% (95 CI −27.7 to −8.9)] at V2. Between treatments, rosuvastatin group had greater reductions at both time points: −1.1 mmol/L at V1 (Tukey-adjusted *p* < 0.001) and −1.0 mmol/L at V2 (Tukey-adjusted *p* < 0.001). The large effect sizes relative to their standard errors (0.21–0.23 mmol/L) corresponded to an achieved power >0.99 at both time points, confirming that the model was adequately powered to detect the observed treatment differences.

**Figure 3:**
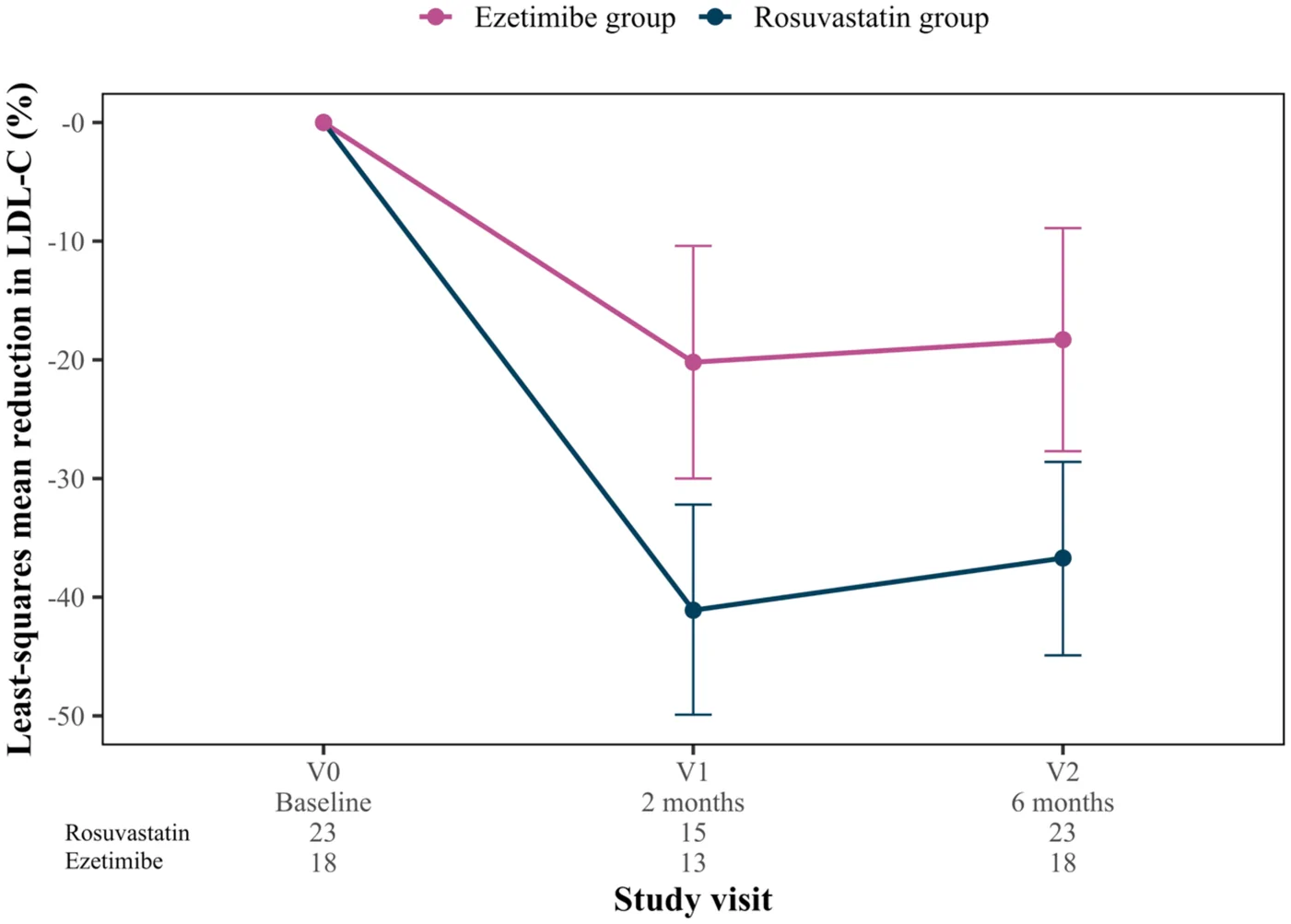
Changes in LDL-C during short-term lipid-lowering therapy (LLT). Adjusted LS mean percentage change in LDL cholesterol from baseline over study visits (V0, V1, and V2) for the rosuvastatin and ezetimibe groups. Error bars indicate 95% confidence intervals. LDL-C, low-density lipoprotein cholesterol; V, study visit.

At the final visit (V2), 13 of 35 children (37.1%) had LDL-C <3.5 mmol/L; 9 of 20 (45%) on rosuvastatin versus 2 of 15 (13%) on ezetimibe achieved this target (Fisher's exact *p* = 0.069; OR = 5.07, 95% CI 0.80–58.0). The analysis was underpowered (61% power), and a larger sample (∼31 per group) would be needed to detect statistical significance. A >50% LDL-C reduction occurred in 4 of 20 rosuvastatin-treated and 0 of 15 ezetimibe-treated children; this difference was also underpowered and not statistically significant (*p* = 0.119), indicating a larger sample is required to confirm significance.

#### 3.1.2 Safety and tolerability

At V1 and V2, no elevations in transaminases or CK were observed. No clinical adverse effects were reported in either the ezetimibe or statin treatment groups during the follow-up period, and no treatment discontinuations due to adverse effects were documented.

### 3.2 Baseline arterial injury markers characteristics

#### 3.2.1 Carotid intima-media thickness (cIMT)

The baseline median cIMT was 0.35 mm (IQR 0.35–0.40; range 0.28–0.65). Ten patients (22.2%) had cIMT >0.40 mm, and two (4.4%) >0.50 mm. No difference in cIMT was observed between males and females (*p* = 0.535). Height *z*-score (*ρ* = 0.588, *p* < 0.001), weight *z*-score (*ρ* = 0.497, *p* < 0.001), and BMI *z*-score (*ρ* = 0.374, *p* = 0.011) correlated positively with cIMT. PWV (*p* = 0.322), RHI (*p* = 0.089), beta stiffness (*p* = 0.180), age (*p* = 0.128), and sex (*p* = 0.541) were not correlated.

Among lipid variables, TC (*p* = 0.531), LDL-C (*p* = 0.279), HDL-C (*p* = 0.575), ApoB (*p* = 0.365), Lp(a) (*p* = 0.534), TC Exp (*p* = 0.259), LDL-C Exp (*p* = 0.813), and ApoB Exp (*p* = 0.074) were not correlated with cIMT. TG correlated positively (*ρ* = 0.500, *p* < 0.001).

In age- and sex-adjusted partial correlations, TG remained correlated with cIMT (*ρ* = 0.571, *p* < 0.001), whereas TC (*p* = 0.481), LDL-C (*p* = 0.958), ApoB (*p* = 0.313), and Lp(a) (*p* = 0.850) remained not correlated.

#### 3.2.2 Pulse wave velocity (PWV)

The median PWV was 3.7 m/s (IQR 3.5–3.9; range 3.1–4.2). Males had higher PWV than females [3.8 (IQR 3.7–4.1) vs. 3.6 (IQR 3.4–3.8); *p* = 0.025]. PWV correlated with beta stiffness (*ρ* = 0.833, *p* < 0.001), age (*ρ* = 0.420, *p* = 0.005), weight *z*-score (*ρ* = 0.417, *p* = 0.005), and BMI *z*-score (*ρ* = 0.466, *p* = 0.001). Height *z*-score (*p* = 0.314), RHI (*p* = 0.450), and cIMT (*p* = 0.322) were not correlated.

Lipid variables showed negative correlations for TC (*ρ* = –0.454, *p* = 0.002), LDL-C (*ρ* = –0.452, *p* = 0.002), and ApoB (*ρ* = –0.423, *p* = 0.009). HDL-C (*p* = 0.821), Lp(a) (*p* = 0.875), TC Exp (*p* = 0.142), LDL-C Exp (*p* = 0.576), ApoB Exp (*p* = 0.443), and TG (*p* = 0.199) were not correlated.

In adjusted analyses, none of the lipid variables correlated with PWV (TC *p* = 0.228; LDL-C *p* = 0.256; ApoB *p* = 0.598; Lp(a) *p* = 0.587; TG *p* = 0.402).

#### 3.2.3 Reactive hyperemia index (RHI)

The median RHI was 1.51 (IQR 1.12–1.82; range 0.85–3.48). Of the 41 participants, 25 (61%) had endothelial dysfunction, 10 (24%) were in the grey zone, and 6 (15%) had normal function. No sex difference was observed (*p* = 0.937). RHI correlated positively with age (*ρ* = 0.585, *p* < 0.001). Height *z*-score (*p* = 0.373), weight *z*-score (*p* = 0.884), BMI *z*-score (*p* = 0.508), sex (*p* = 0.938), PWV (*p* = 0.450), cIMT (*p* = 0.089), and beta stiffness (*p* = 0.203) were not correlated.

For lipid variables, negative correlations were observed for TC (*ρ* = –0.515, *p* < 0.001), LDL-C (*ρ* = –0.582, *p* < 0.001), and ApoB (*ρ* = –0.622, *p* < 0.001). TC Exp (*ρ* = 0.482, *p* = 0.001), LDL-C Exp (*ρ* = 0.351, *p* = 0.025), and ApoB Exp (*ρ* = 0.321, *p* = 0.041) correlated positively with RHI. HDL-C (*p* = 0.278), Lp(a) (*p* = 0.837), and TG (*p* = 0.596) were not correlated.

After adjustment for age and sex, LDL-C (*ρ* = –0.348, *p* = 0.030) and ApoB (*ρ* = –0.385, *p* = 0.029) remained correlated with RHI. However, an estimated 63 participants would be required for these effect sizes to reach 80% statistical power. TC (*p* = 0.176), Lp(a) (*p* = 0.607), and TG (*p* = 0.764) were not correlated.

#### 3.2.4 Beta stiffness

Median beta stiffness was 3.2 (IQR 2.8–3.6; range 2.1–4.9). Beta stiffness correlated positively with age (*ρ* = 0.374, *p* = 0.012). Height *z*-score (*p* = 0.406), weight *z*-score (*p* = 0.177), BMI *z*-score (*p* = 0.080), sex (*p* = 0.348), RHI (*p* = 0.203), and cIMT (*p* = 0.180) were not correlated.

Negative correlations were observed for TC (*ρ* = –0.419, *p* = 0.005), LDL-C (*ρ* = –0.447, *p* = 0.002), and ApoB (*ρ* = –0.397, *p* = 0.015). HDL-C (*p* = 0.699), Lp(a) (*p* = 0.445), TC Exp (*p* = 0.197), LDL-C Exp (*p* = 0.862), ApoB Exp (*p* = 0.668), and TG (*p* = 0.380) were not correlated.

Adjusted analyses showed no correlations between beta stiffness and lipid variables (TC *p* = 0.167; LDL-C *p* = 0.097; ApoB *p* = 0.283; Lp(a) *p* = 0.217; TG *p* = 0.597).

### 3.3 Changes in arterial injury markers during the treatment period

At V2, arterial injury markers were successfully obtained in 34 patients for cIMT, PWV, and beta stiffness, and in 31 patients for RHI.

Arterial injury markers at V2 and their changes from V1 are presented in Figure 4 and Supplementary Table S1. Two ANCOVA models for each marker were conducted, the overall model with baseline cIMT and LDL-C change between the visits and second, treatment group model with the addition of treatment group (rosuvastatin group and ezetimibe group).

**Figure 4:**
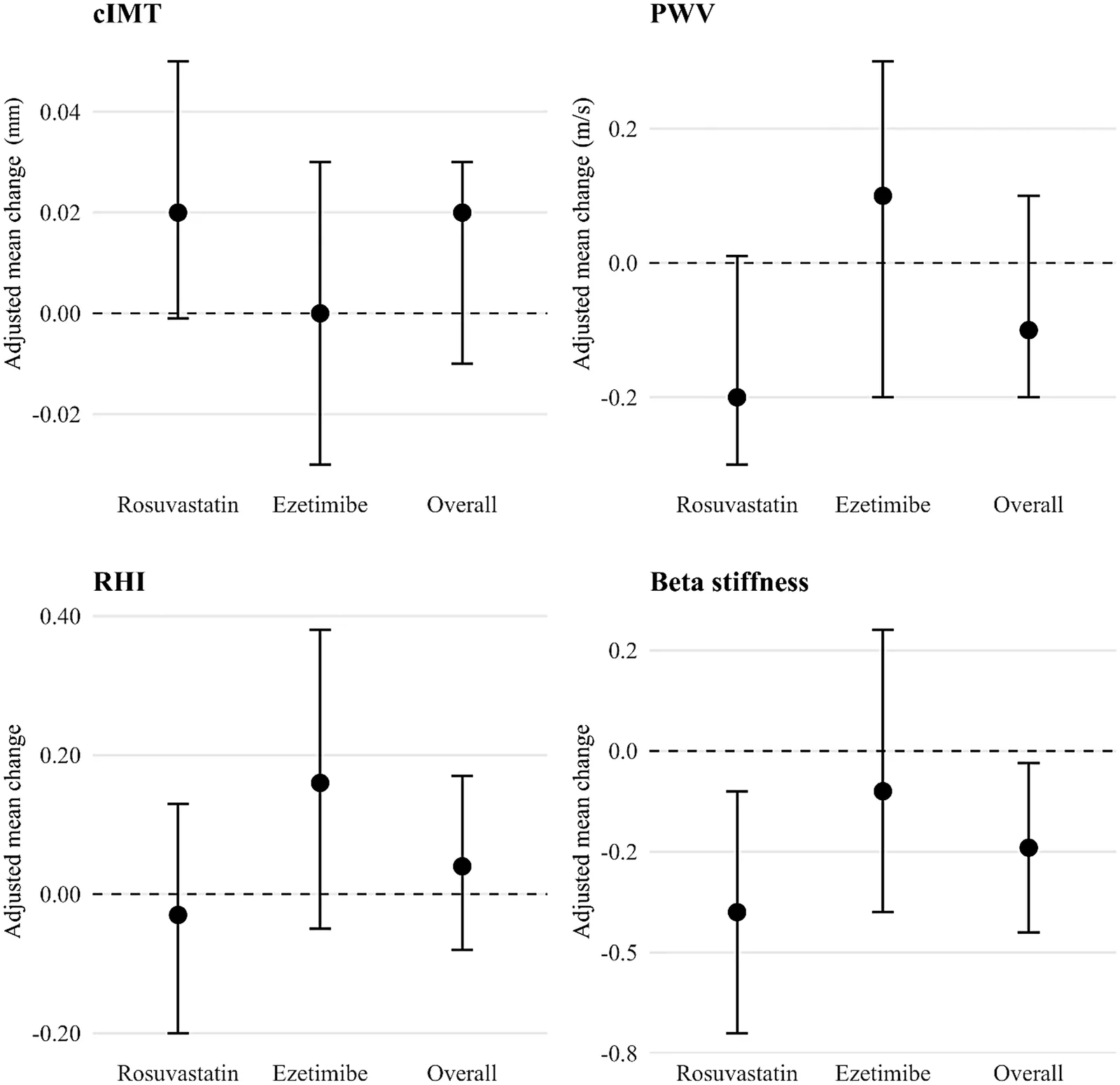
Adjusted mean changes in carotid intima–media thickness (cIMT), pulse wave velocity (PWV), reactive hyperaemia index (RHI), and beta stiffness following short-term lipid-lowering therapy with rosuvastatin or ezetimibe. Values represent estimated marginal means (EMM). Adjusted mean changes with 95% confidence intervals were derived from ANCOVA models including only baseline marker value and LDL-C reduction. Treatment-group EMMs (rosuvastatin and ezetimibe) were obtained from extended models that additionally included treatment allocation as a fixed factor. All values shown are EMMs with 95% confidence intervals. If the 95% CI included 0, the EMM change was regarded as non-detectable in our cohort. Across all vascular markers, baseline marker values consistently showed the largest effect sizes and were the only predictors with sufficient power for detection in our sample (partial *η*^2^ ≈ 0.12–0.36). By contrast, LDL-C reduction (partial *η*^2^ ≤ 0.05) and treatment-group contrasts (rosuvastatin vs. ezetimibe) (partial *η*^2^ 0.04–0.07) exhibited small effect sizes and were therefore underpowered. The magnitude of observed EMM for beta stiffness (0.1–0.3 units) is not clinically meaningful. cIMT, carotid intima-media thickness; PWV, pulse wave velocity; RHI, reactive hyperaemia index; mm, millimetres; m/s, metres per second.

At V2, cIMT median was 0.38 mm (95% CI 0.35–0.40) in the rosuvastatin group, 0.37 mm (95% CI 0.34–0.39) in the ezetimibe group, and 0.37 mm (95% CI 0.35–0.39) overall. PWV 3.6 m/s (95% CI 3.4–3.8) in the rosuvastatin and 3.8 m/s (95% CI 3.6–4.0) in ezetimibe group, with an overall median of 3.7 m/s (95% CI 3.6–3.8). RHI median was 1.49 (95% CI 1.34–1.65) in the rosuvastatin and 1.72 (95% CI 1.54–1.90) in ezetimibe group, with a combined median of 1.58 (95% CI 1.45–1.72). Beta stiffness median was 3.1 (95% CI 2.8–3.3) in the rosuvastatin group and 3.1 (95% CI 2.8–3.4) in the ezetimibe group, with an overall median at V2 of 3.1 (95% CI 2.9–3.3).

#### 3.3.1 Carotid intima–media thickness (cIMT)

In the whole-cohort ANCOVA model for cIMT change, neither LDL-C reduction (*p* = 0.402; partial *η*^2^ = 0.023) nor baseline cIMT (*p* = 0.051; partial *η*^2^ = 0.118) was associated with cIMT change. The adjusted mean cIMT change for the cohort was +0.02 mm (95% CI −0.01 to +0.03), and detecting an LDL-C effect of this magnitude would require approximately 330 participants. In the treatment-adjusted model, LDL-C reduction again remained non-significant (*p* = 0.212; partial *η*^2^ = 0.051), whereas baseline cIMT was significantly associated with change (*B* = –0.410 mm; 95% CI −0.799 to −0.021; *p* = 0.040; partial *η*^2^ = 0.134). Group allocation did not predict cIMT change (rosuvastatin vs. ezetimibe: +0.02 mm; 95% CI −0.20 to +0.65; *p* = 0.287; partial *η*^2^ = 0.038), and detecting a difference of this magnitude would require approximately 200 participants. Adjusted mean changes were +0.02 mm (95% CI −0.00 to +0.05) in the rosuvastatin group and 0.00 mm (95% CI −0.03 to +0.03) in the ezetimibe group.

#### 3.3.2 Pulse wave velocity (PWV)

In the overall PWV model, LDL-C reduction was not significantly associated with PWV change (*p* = 0.517; partial *η*^2^ = 0.014), whereas baseline PWV showed a borderline association (*B* = –0.469 m/s; 95% CI −0.941 to +0.003; *p* = 0.051; partial *η*^2^ = 0.117). The adjusted mean PWV change for the cohort was −0.1 m/s (95% CI −0.2 to +0.1), and detecting an LDL-C effect of this magnitude would require approximately 550 participants. In the treatment-adjusted model, LDL-C reduction again remained non-significant (*p* = 0.195; partial *η*^2^ = 0.055), and baseline PWV continued to be the strongest continuous predictor (*B* = –0.384 m/s; 95% CI −0.864 to +0.096; *p* = 0.113; partial *η*^2^ = 0.082). The difference between rosuvastatin and ezetimibe was small and non-significant (–0.2 m/s; 95% CI −0.534 to +0.094; *p* = 0.163), with adjusted mean changes of −0.2 m/s (95% CI −0.3 to +0.0) and +0.1 m/s (95% CI −0.2 to +0.3), respectively. Detecting the group difference would require approximately 110.

#### 3.3.3 Reactive hyperemia index (RHI)

In the overall RHI model, LDL-C reduction showed a trend toward association with RHI change (*p* = 0.094; partial *η*^2^ = 0.097), while baseline RHI was a strong predictor (*B* = –0.625; 95% CI −0.951 to −0.300; *p* = 0.001; partial *η*^2^ = 0.356). The adjusted mean RHI change for the cohort was +0.04 (95% CI −0.08 to +0.17), and detecting an LDL-C effect of this magnitude would require approximately 70 participants. In the treatment-adjusted model, LDL-C reduction was not associated with RHI change (*p* = 0.394; partial *η*^2^ = 0.027), whereas baseline RHI remained strongly predictive (*B* = –0.618; 95% CI −0.939 to −0.297; *p* = 0.001; partial *η*^2^ = 0.366). The rosuvastatin–ezetimibe difference was small and non-significant (–0.20; 95% CI −0.49 to +0.95; *p* = 0.178; partial *η*^2^ = 0.066), with approximately 110 participants needed to detect an effect of this magnitude. Adjusted mean changes were −0.03 (95% CI −0.20 to +0.13) with rosuvastatin and +0.16 (95% CI −0.05 to +0.38) with ezetimibe.

#### 3.3.4 Beta stiffness

In the overall beta stiffness model, LDL-C reduction was not associated with change (*p* = 0.824; partial *η*^2^ = 0.002), whereas baseline beta stiffness was strongly associated with subsequent change (*B* = –0.783; 95% CI −1.165 to −0.402; *p* < 0.001; partial *η*^2^ = 0.362). The adjusted mean change in beta stiffness for the cohort was −0.24 (95% CI −0.45 to −0.03; *p* = 0.002). Given the very small effect size for LDL-C (partial *η*^2^ = 0.002), detecting an LDL-C–related effect of this magnitude would require an estimated 3,900 participants. In the treatment-adjusted model, LDL-C reduction again was not a significant predictor (*p* = 0.419; partial *η*^2^ = 0.022), while baseline beta stiffness remained the dominant continuous predictor (*p* = 0.001; partial *η*^2^ = 0.296). Treatment allocation did not significantly predict beta stiffness change (rosuvastatin vs. ezetimibe: −0.3; 95% CI −0.8 to +0.2; *p* = 0.266; partial *η*^2^ = 0.041), and detecting a difference of this magnitude would require approximately 180 participants. Adjusted mean changes were −0.4 (95% CI −0.7 to −0.1; *p* = 0.028) with rosuvastatin and −0.1 (95% CI −0.4 to +0.3) with ezetimibe.

## 4 Discussion

In this prospective, open-label, randomised study, we present the first head-to-head comparison of rosuvastatin and ezetimibe in treatment-naïve children with heterozygous familial hypercholesterolaemia (HeFH) and systematically assess the safety and tolerability of both therapies. In addition, we conducted the first integrated evaluation of multiple non-invasive arterial injury markers—including carotid intima-media thickness (cIMT), pulse wave velocity (PWV), reactive hyperemia index (RHI), and beta stiffness—characterising their baseline profiles and short-term trajectories.

We found that first-line treatment with 5 mg once-daily rosuvastatin led to greater and faster LDL-C reduction than 10 mg once-daily ezetimibe during short-term lipid-lowering therapy. These findings confirm the effectiveness of lipid-lowering therapy in children with familial hypercholesterolaemia and support the superior LDL-C–lowering efficacy of statins compared with ezetimibe when used as monotherapy (15). Notably, the magnitude of these differences was large relative to their standard errors, providing excellent statistical power to detect both the overall LDL-C decrease and the between-treatment contrasts, despite the modest sample size. A greater proportion of children treated with rosuvastatin achieved LDL-C below 3.5 mmol/L compared with those receiving ezetimibe (36% vs. 13%), although this comparison was underpowered due to the small sample sizes and the low number of events in the ezetimibe group. Similarly, we could not demonstrate a statistically significant difference in the proportion achieving greater than 50% LDL-C reduction, despite 20% of children in the rosuvastatin group reaching this response compared with none in the ezetimibe group. Importantly, baseline LDL-C levels were high in both groups, which should be considered when interpreting response thresholds.

Known adverse effects of rosuvastatin in children include muscle-related symptoms and abnormalities in laboratory measurements (16, 17). A 20-year follow-up study of 214 patients with FH who began statin therapy at a median age of 14 years reported no serious adverse events and no differences in liver function tests or creatine kinase levels compared with unaffected siblings (18). In our cohort, rosuvastatin was started at a younger median age of 9.7 years, with no reports of myalgia or other adverse effects, and no elevations in liver transaminases or creatine kinase detected during short-term lipid-lowering therapy. Adverse effects of ezetimibe are generally uncommon and mild, with transient diarrhea among the most frequently reported adverse effects (19). In our study, no children receiving ezetimibe discontinued treatment or reported treatment-related adverse effects. Similarly, no children receiving statin therapy discontinued treatment or reported treatment-related adverse effects. Thus, no apparent differences in tolerability between ezetimibe and statin therapy were observed in our cohort, although the relatively small sample size limits the ability to detect uncommon adverse events.

We observed a positive correlation between cIMT and anthropometric *z*-scores, suggesting that variation in cIMT during childhood is largely driven by growth- and puberty-related hemodynamic changes rather than chronological age alone (20). Sex- and age-related differences may become more apparent later in adolescence (21–23), which could explain their absence in our cohort. Although previous studies have identified LDL-C and the presence of FH, independently of LDL-C levels, as predictors of increased cIMT, we did not observe such an association (24, 25). LDL-C showed only a weak correlation with cIMT, and our sample size was likely insufficient to detect effects of this magnitude with adequate statistical power. Supporting our findings, cIMT progression in childhood may not be accelerated due to FH as historically expected. Reported annual increases in cIMT among healthy children range from approximately 3–10 µm, and even in studies showing greater progression in FH children, values largely remained within this range (22, 24, 26–28).

PWV increased with age and weight *z*-score, consistent with reports that weight is an important predictor of PWV increase (29). We also observed higher PWV in males, consistent with observations of earlier and greater increases in boys than in girls, likely reflecting pubertal development and corresponding changes in the arterial collagen-to-elastin ratio (10, 30). Beta stiffness closely mirrored PWV, as it increased with age but showed no sex differences and, as expected, proved to be less dependent on body size compared to PWV (31). Neither stiffness marker was associated with lipid variables when adjusted for age and sex. However, the study was underpowered to detect such small effects of lipids, further limiting the interpretability of these associations. When we compared baseline PWV or beta stiffness in our cohort with healthy controls assessed using the same echo-tracking method, values were in the lower range of those reported by Jurko et al., indicating no difference between FH and healthy children (32). Additionally, recent reports suggest that PWV is not markedly altered in either pediatric or adult populations with FH (33).

Endothelial function was assessed using RHI measured by the EndoPAT device. RHI increased with age but was not associated with standardised anthropometric variables, suggesting that factors beyond somatic growth influence endothelial function, as changes in endothelial function during childhood are likely driven by multiple processes, including pubertal maturation, alterations in vascular homeostasis, and shifts in insulin sensitivity (8, 34, 35). LDL-C and ApoB showed a strong inverse correlation with RHI, and importantly, the association with LDL-C remained significant after adjustment for age and sex, although the available sample size was insufficient to evaluate this relationship with adequate precision. A larger cohort would therefore be required to confirm the robustness of this association. This finding is consistent with a meta-analysis by Urbina et al., which demonstrated that children with FH exhibit measurable endothelial dysfunction compared with age- and sex-matched controls (7, 36). Additionally, nearly two-thirds of our cohort had RHI values indicative of endothelial dysfunction; however, Radtke et al. demonstrated that most healthy prepubertal children would be classified as having endothelial dysfunction if adult thresholds were applied, highlighting the need for age-appropriate reference values (37).

When analysing short-term changes in arterial injury markers, only beta stiffness showed a statistically significant decrease. This significant reduction was observed in the cohort as a whole and within the rosuvastatin group, but not in the ezetimibe group. Importantly, this change was predicted solely by baseline beta stiffness and not by LDL-C reduction or treatment allocation, a pattern most consistent with regression towards the mean (38). The adjusted between-group difference in beta stiffness change was not statistically significant, likely reflecting limited statistical power and a small underlying effect size; therefore, larger cohorts and longer follow-up may be required to detect any true treatment-related differences. Moreover, although the adjusted changes in beta stiffness reached statistical significance (approximately 0.1–0.3 units), their magnitude was small and unlikely to be clinically meaningful (39). Across all markers (cIMT, PWV, RHI, and beta stiffness), the effects of LDL-C reduction or treatment group were uniformly small. *Post hoc* power analyses confirmed that all vascular models were underpowered to detect LDL-C–dependent or treatment-specific effects, and substantially larger samples would be required to identify such associations reliably. The short follow-up interval further compounded this limitation, as structural arterial markers typically change slowly, and detecting subtle short-term effects requires much larger cohorts. Although short-term improvements have been reported in some studies, these were conducted in adults, who exhibit less growth-related vascular variability, or in considerably larger samples, making such effects more detectable (18, 40). Although some studies have reported short-term improvements, these were conducted in adults who have less confounding growth-related vascular variability or involved substantially larger cohorts, making such effects more detectable (1–3). For example, a recent study in adults showed that adding a PCSK9 inhibitor to high-intensity statin plus ezetimibe therapy improved pulse wave velocity (PWV) after 6 months of treatment, suggesting that newer lipid-lowering therapies may provide additional vascular benefits (41). In children, strong influences of growth, interindividual differences in pubertal maturation, physical activity, lifestyle factors, obesity, and diabetes can all obscure potential effects of dyslipidemia treatment (17, 42–44). However, we aimed to minimise these influences by enrolling only children with normal weight, height, and BMI for age, none of whom had arterial hypertension or diabetes, thereby reducing the impact of comorbid cardiovascular risk factors on vascular assessments. Finally, the sensitivity of the arterial injury markers used in our study was originally optimised for adult vascular physiology and may be insufficient to capture the subtle and variable early arterial changes occurring in children over a short follow-up interval (10, 23, 45–47). Alternative methods may provide greater sensitivity for short-term vascular changes, as suggested in recent studies (23, 45, 46).

This study has several limitations. The cohort was relatively small, and 22% of children did not complete the final follow-up assessment, due to missed appointments rather than treatment-related concerns. To our knowledge, none of the children who were lost to follow-up reported treatment-related adverse effects, either with rosuvastatin or ezetimibe, suggesting that treatment intolerance was unlikely to be a major reason for loss to follow-up. Nevertheless, sex, treatment group, baseline height *z*-score, and LDL-C did not differ between completers and non-completers, indicating that dropout is unlikely to have caused major bias, although those lost to follow-up had slightly higher baseline weight and BMI *z*-scores. Additionally, all included children were advised to follow a healthy, balanced diet with limited intake of animal-derived fats and adequate consumption of vegetables and polyunsaturated fats. However, dietary intake and adherence to these recommendations were not systematically assessed and therefore could not be accounted for in the analysis. The treatment groups were not matched or adjusted for pubertal stage, and differences in sexual maturation may therefore have influenced the observed results. Finally, the relatively short follow-up duration, limits conclusions regarding arterial injury markers, as structural and functional vascular changes may evolve gradually and true physiological or treatment-related effects may not yet be detectable over this time period in growing children. Longer-term follow-up is therefore needed to determine whether these markers change over time and whether early treatment effects translate into sustained vascular benefit. Overall, the study was sufficiently powered to detect overall LDL-C reduction and differences between rosuvastatin and ezetimibe efficacy, but was underpowered for weaker baseline analysis, treatment goal achievement, and most analyses involving vascular marker changes, especially the effect of LDL-C reduction or treatment allocation. Finally, we did not include an untreated control group because the primary objective was to compare two established LDL-C-lowering therapies, and an open-label design was appropriate given that all primary endpoints were objectively assessed.

## 5 Conclusions

This study is the first head-to-head randomized trial demonstrating greater short-term LDL-C reduction with rosuvastatin compared with ezetimibe (37% vs. 18%), with 45% of children achieving LDL-C <3.5 mmol/L in the rosuvastatin group and 13% in the ezetimibe group. Importantly, no differences in safety or tolerability were observed, as no adverse events occurred in either group. Taken together, these findings support the short-term efficacy and tolerability of rosuvastatin in pediatric patients. All anaysed arterial injury markers (cIMT, PWV, RHI and beta stiffness) were predominantly correlated by age or anthropometry and only RHI showed relevant correlations with lipids. During the short-term LTT, changes in arterial injury markers were unrelated to treatment allocation or LDL-C reduction, although our analyses were underpowered to detect such small effects and larger cohorts or longer follow-up would be needed to evaluate these associations with greater sensitivity.

## Data Availability

The data supporting the findings of this study are openly available in Mendeley Data at https://data.mendeley.com/datasets/knbx56fvz5/1 (doi: 10.17632/knbx56fvz5.1).
