# Supplementary material for: Effect of rosuvastatin and ezetimibe therapy on LDL-cholesterol reduction and non-invasive arterial injury markers in children with heterozygous familial hypercholesterolemia: a prospective, open-label, randomized study
Source: Front Cardiovasc Med. 2026 Sep 29;13:1933757. doi: 10.3389/fcvm.2026.1933757 (PMC13657965; doi:10.3389/fcvm.2026.1933757)
Supplement: Supplementary file 1 [file Datasheet1.docx]

# **SUPPLEMENTARY MATERIALS**

**Figure S1: Correlation Heatmap showing Spearman partial correlation coefficients between arterial injury markers and clinical characteristics at baseline (V0). Red colour indicates positive, while blue indicates negative correlation coefficient between listed variables; Significant correlations are bolded (p<0.05).** Abbreviations: cIMT – Carotid Intima-Media Thickness; PWV – Pulse Wave Velocity; RHI – Reactive Hyperemia Index; BMI – Body Mass Index; TC – Total Cholesterol; ApoB – Apolipoprotein B; LDL-C – Low-Density Lipoprotein Cholesterol; TC Exp – Cumulative TC Exposure; ApoB Exp – Cumulative ApoB Exposure; LDL Exp – Cumulative LDL-C Exposure; Lp(a) – Lipoprotein(a); TG – Triglycerides.

| Arterial injury marker | n | Model | Covatiates | Fixed factor (rosuvastatin vs ezetimibe) | EMM change (95 CI) | Required sample size for ≥0.80 |
| --- | --- | --- | --- | --- | --- | --- |
| cIMT, overall model | 34 | F(2,31) = 2.154, p = 0.133; R² = 0.122; adjusted R² = 0.065; power = 0.407 | LDL-C reduction (B = 0.008, 95% CI –0.011 to +0.027; p = 0.402; partial η² = 0.023);  Baseline cIMT (B = –0.386, 95% CI –0.773 to +0.001; p = 0.051; partial η² = 0.118) | / | +0.02 (–0.01 to +0.03) | LDL-C effect: n ≈ 330 |
| cIMT, treatment-group model | 34 | F(3,30) = 1.836, p = 0.162; R² = 0.155; adjusted R² = 0.071; power = 0.428 | LDL-C reduction (B = 0.013, 95% CI –0.008 to +0.035; p = 0.212; partial η² = 0.051);  Baseline cIMT (B = –0.410, 95% CI –0.799 to –0.021; p = 0.040; partial η² = 0.134) | B = 0.023, 95% CI –0.020 to +0.065; p = 0.287; partial η² = 0.038 | Rosu: +0.02 (–0.00 to +0.05); Eze: 0.00 (–0.03 to +0.03) | LDL-C effect: n ≈ 330; group difference: n ≈ 200 |
| PWV, overall model | 34 | F(2,31) = 3.225, p = 0.053; R² = 0.172; adjusted R² = 0.119; power = 0.573 | LDL-C reduction (B = –0.046, 95% CI –0.189 to +0.097; p = 0.517; partial η² = 0.014);  Baseline PWV (B = –0.469, 95% CI –0.941 to +0.003; p = 0.051; partial η² = 0.117) | / | –0.1 m/s (–0.2 to +0.1), | LDL-C effect: n ≈ 550 |
| PWV, treatment-group model | 34 | F(3,30) = 2.906, p = 0.051; R² = 0.225; adjusted R² = 0.148; power = 0.633 | LDL-C reduction (B = –0.108, 95% CI –0.274 to +0.058; p = 0.195; partial η² = 0.055);  Baseline PWV (B = –0.384, 95% CI –0.864 to +0.096; p = 0.113; partial η² = 0.082) | B = –0.220, 95% CI –0.534 to +0.094; p = 0.163; partial η² = 0.064 | Rosu: –0.2 (–0.3 to +0.0); Eze: +0.1 (–0.2 to +0.3) | LDL-C effect: n ≈ 130; group difference: n ≈ 110 |
| RHI, overall model | 31 | F(2,28) = 7.820, p = 0.002; R² = 0.358; adjusted R² = 0.313; power = 0.928 | LDL-C reduction (B = 0.110, 95% CI –0.020 to +0.241; p = 0.094; partial η² = 0.097);  Baseline RHI (B = –0.625, 95% CI –0.951 to –0.300; p = 0.001; partial η² = 0.356) | / | +0.04 (–0.08 to +0.17) | LDL-C effect: n ≈ 70 |
| beta stiffness, overall model | 34 | F(2,31) = 9.993, p < 0.001; R² = 0.392; adjusted R² = 0.353; power = 0.975 | LDL-C reduction(B = –0.024, 95% CI –0.247 to +0.198; p = 0.824; partial η² = 0.002),  Baseline stiffness(B = –0.783, 95% CI –1.165 to –0.402; p < 0.001; partial η² = 0.362) | / | –0.24 (–0.45 to –0.03), p=0.002 | LDL-C effect: n ≈ 3900 |
| Beta stiffness, treatment group model | 34 | F(3,30) = 7.150, p = 0.001; R² = 0.417; adjusted R² = 0.359; power = 0.966 | LDL-C reduction (B = –0.107, 95% CI –0.375 to +0.160; p = 0.419; partial η² = 0.022);  Baseline stiffness (B = –0.705, 95% CI –1.110 to –0.300; p = 0.001; partial η² = 0.296) | B = –0.291, 95% CI –0.816 to +0.234; p = 0.266; partial η² = 0.041). | Rosu: –0.4 (–0.7 to –0.1); Eze: –0.1 (–0.4 to +0.3) | LDL-C reduction n≈ 1200; treatment n≈ 180 |

**Table S1.** Summary of ANCOVA Models Evaluating Predictors of Short-Term Change in Vascular Markers. ANCOVA models were constructed to examine the association between LDL-C reduction, baseline vascular marker values, and treatment allocation (rosuvastatin vs. ezetimibe) with changes in arterial injury markers. For each model, degrees of freedom (df), F-statistics, p-values, variance explained (R² and adjusted R²), observed power, regression coefficients (B) with 95% confidence intervals (CIs), partial eta-squared (η²), adjusted mean changes with 95% CIs, and estimated sample sizes required to detect the observed effect sizes with 80% power are reported. Abbreviations: EMM - Estimated Marginal Mean; cIMT – Carotid Intima-Media Thickness; PWV – Pulse Wave Velocity; RHI – Reactive Hyperemia Index; LDL-C – Low-Density Lipoprotein; Cholesterol; Rosu — Rosuvastatin; Eze — Ezetimibe
